# (*E*)-1-[2-(4-Chloro-2-nitro­styr­yl)-1-phenyl­sulfonyl-1*H*-indol-3-yl]propan-1-one

**DOI:** 10.1107/S1600536813031073

**Published:** 2013-11-16

**Authors:** M. Umadevi, V. Saravanan, R. Yamuna, A. K. Mohanakrishnan, G. Chakkaravarthi

**Affiliations:** a Research Scholar (Chemistry), Bharathiyar University, Coimbatore 641 046, Tamilnadu, India; b Department of Organic Chemistry, University of Madras, Guindy Campus, Chennai 600 025, India; c Department of Sciences, Chemistry and Materials Research Lab, Amrita Vishwa Vidyapeetham University, Ettimadai, Coimbatore 641 112, India; d Department of Physics, CPCL Polytechnic College, Chennai 600 068, India

## Abstract

In the title compound, C_25_H_19_ClN_2_O_5_S, the phenyl ring forms dihedral angles of 79.62 (12) and 80.02 (13)° with the indole ring system and the benzene ring, respectively. The nitro group is twisted at an angle of 22.39 (11)° with respect to the attached benzene ring. In the crystal, mol­ecules assemble into double layers in the *ab* plane *via* C—H⋯O inter­actions.

## Related literature

For the biological activity of indole derivatives, see: Okabe & Adachi (1998 ▶); Srivastava *et al.* (2011 ▶). For related structures, see: Chakkaravarthi *et al.* (2008 ▶, 2010 ▶).
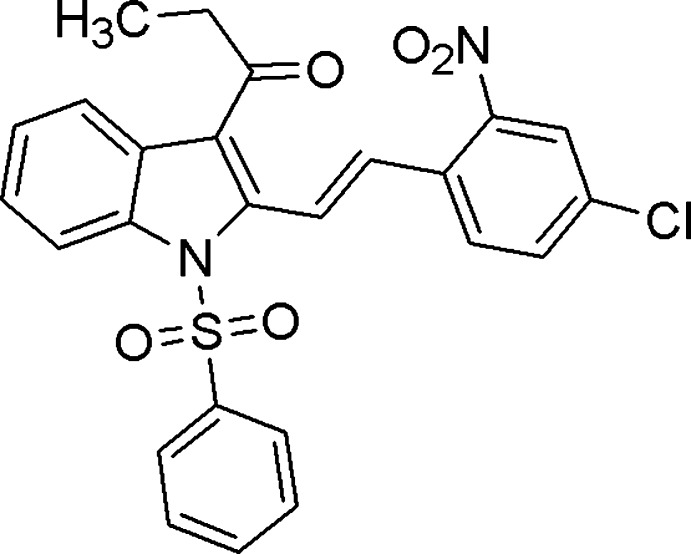


## Experimental

### 

#### Crystal data

C_25_H_19_ClN_2_O_5_S
*M* *_r_* = 494.93
Triclinic, 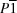

*a* = 8.4658 (3) Å
*b* = 8.6643 (3) Å
*c* = 16.1126 (6) Å
α = 84.196 (2)°
β = 87.768 (3)°
γ = 79.541 (2)°
*V* = 1156.01 (7) Å^3^
*Z* = 2
Mo *K*α radiation
μ = 0.30 mm^−1^
*T* = 295 K
0.28 × 0.24 × 0.20 mm

#### Data collection

Bruker Kappa APEXII diffractometer
Absorption correction: multi-scan (*SADABS*; Sheldrick, 1996 ▶) *T*_min_ = 0.922, *T*_max_ = 0.943
25531 measured reflections
6333 independent reflections
4536 reflections with *I* > 2σ(*I*)
*R*_int_ = 0.029

#### Refinement

*R*[*F* ^2^ > 2σ(*F* ^2^)] = 0.050
*wR*(*F* ^2^) = 0.150
*S* = 1.03
6333 reflections
308 parameters
H-atom parameters constrained
Δρ_max_ = 0.49 e Å^−3^
Δρ_min_ = −0.39 e Å^−3^

### 

Data collection: *APEX2* (Bruker, 2004 ▶); cell refinement: *SAINT* (Bruker, 2004 ▶); data reduction: *SAINT*; program(s) used to solve structure: *SHELXS97* (Sheldrick, 2008 ▶); program(s) used to refine structure: *SHELXL97* (Sheldrick, 2008 ▶); molecular graphics: *PLATON* (Spek, 2009 ▶); software used to prepare material for publication: *SHELXL97*.

## Supplementary Material

Crystal structure: contains datablock(s) I, global. DOI: 10.1107/S1600536813031073/tk5271sup1.cif

Structure factors: contains datablock(s) I. DOI: 10.1107/S1600536813031073/tk5271Isup2.hkl

Supplementary material file. DOI: 10.1107/S1600536813031073/tk5271Isup3.cml

Additional supplementary materials:  crystallographic information; 3D view; checkCIF report

## Figures and Tables

**Table 1:** Hydrogen-bond geometry (Å, °)

*D*—H⋯*A*	*D*—H	H⋯*A*	*D*⋯*A*	*D*—H⋯*A*
C10—H10⋯O1^i^	0.93	2.60	3.327 (3)	136
C16—H16*B*⋯O5^ii^	0.97	2.37	3.260 (3)	152

Symmetry codes: (i) 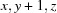; (ii) 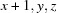.

**Fig. 1.**
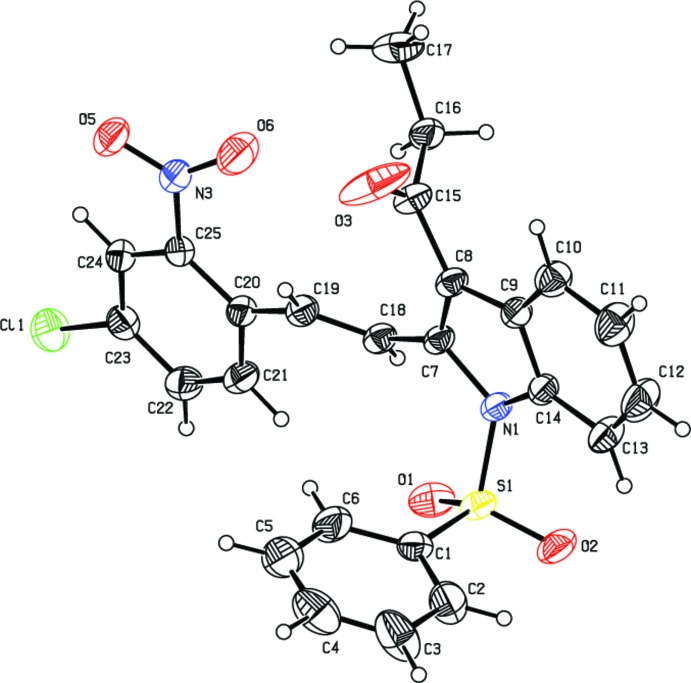
The molecular structure of (I), with atom labels and 30% probability displacement ellipsoids for non-H atoms.

## supplementary crystallographic information

### 2.1. Synthesis and crystallization

A solution of 1-(2-(bromo­methyl)-1-(phenyl­sulfonyl)-1*H*-indol-3-yl)propan-1-one (5 g, 12.31 mmol) and tri­phenyl­phosphine (3.5 g, 13.54 mmol) in dry THF (100 ml) was refluxed for 6 h. After consumption of the starting material, the solvent was removed under vacuum and the solid washed with di­ethyl ether to give the phospho­nium salt. Then, the mixture of phospho­nium salt (8 g, 11.97 mmol), 4-chloro-2-nitro­benzaldehyde (2.45 g, 13.17 mmol) and K_2_CO_3_ (3.30 g, 23.95 mmol) in DCM (70 ml) was stirred at room temperature for 24 h. After completion of the reaction (monitored by TLC), it was diluted using DCM (30 ml), washed with water (2 *x* 100 ml) and dried (Na_2_SO_4_). Removal of solvent *in vacuo* followed by trituration of the crude product with MeOH (20 ml) afforded the title compound.

### 2.2. Refinement

Crystal data, data collection and structure refinement details are summarized in Table 1. H atoms were positioned geometrically and refined using riding model, with C—H = (0.93–0.97) Å and *U*_iso_(H) = 1.2–1.5*U*_eq_(C). Owing to poor agreement, the (001) reflection was omitted from the final cycles of refinement.

### 3. Results and discussion

In continuation of our studies on indole derivatives which are known to exhibit anti-microbial, anti-biotic, analgesic and anti-cancer activities (Okabe and Adachi, 1998; Srivastava *et al.*, 2011), we herewith report the crystal structure of the title compound (I). The geometric parameters of (I) (Fig. 1) are in close agreement with similar structures (Chakkaravarthi *et al.*, 2008; 2010).

The phenyl ring makes the dihedral angle of 79.62 (12)° with the indole ring system. The phenyl ring (C1—C6) and the benzene ring (C20—C25) are inclined at an angle of 80.02 (13)°. The nitro group is twisted at an angle of 22.39 (11)° with respect to the attached benzene ring (C20—C25). The N1 atom is *sp*^2^ hybridised as the bond angles around N1 atom sum 352.4°. Details of the C—H···O inter­actions are given in Table 2 - these lead to layers in the *ab* plane.

### Crystal data

**Table:**

C_25_H_19_ClN_2_O_5_S	*Z* = 2
*M**_r_* = 494.93	*F*(000) = 512
Triclinic, *P*1	*D*_x_ = 1.422 Mg m^−^^3^
Hall symbol: -P 1	Mo *K*α radiation, λ = 0.71073 Å
*a* = 8.4658 (3) Å	Cell parameters from 8917 reflections
*b* = 8.6643 (3) Å	θ = 2.5–28.4°
*c* = 16.1126 (6) Å	µ = 0.30 mm^−^^1^
α = 84.196 (2)°	*T* = 295 K
β = 87.768 (3)°	Block, colourless
γ = 79.541 (2)°	0.28 × 0.24 × 0.20 mm
*V* = 1156.01 (7) Å^3^	

### Data collection

**Table:**

Bruker Kappa APEXII diffractometer	6333 independent reflections
Radiation source: fine-focus sealed tube	4536 reflections with *I* > 2σ(*I*)
Graphite monochromator	*R*_int_ = 0.029
ω and φ scan	θ_max_ = 29.5°, θ_min_ = 2.4°
Absorption correction: multi-scan (*SADABS*; Sheldrick, 1996)	*h* = −11→11
*T*_min_ = 0.922, *T*_max_ = 0.943	*k* = −11→11
25531 measured reflections	*l* = −20→22

### Refinement

**Table:**

Refinement on *F*^2^	Primary atom site location: structure-invariant direct methods
Least-squares matrix: full	Secondary atom site location: difference Fourier map
*R*[*F*^2^ > 2σ(*F*^2^)] = 0.050	Hydrogen site location: inferred from neighbouring sites
*wR*(*F*^2^) = 0.150	H-atom parameters constrained
*S* = 1.03	*w* = 1/[σ^2^(*F*_o_^2^) + (0.0709*P*)^2^ + 0.3517*P*] where *P* = (*F*_o_^2^ + 2*F*_c_^2^)/3
6333 reflections	(Δ/σ)_max_ < 0.001
308 parameters	Δρ_max_ = 0.49 e Å^−^^3^
0 restraints	Δρ_min_ = −0.39 e Å^−^^3^

### Fractional atomic coordinates and isotropic or equivalent isotropic displacement parameters (Å^2^)

**Table:**

	*x*	*y*	*z*	*U*_iso_*/*U*_eq_	
C1	0.6620 (2)	0.1560 (2)	0.44142 (11)	0.0497 (4)	
C2	0.7468 (3)	0.1501 (4)	0.51342 (14)	0.0765 (7)	
H2	0.8570	0.1131	0.5140	0.092*	
C3	0.6662 (4)	0.1997 (5)	0.58429 (16)	0.0984 (11)	
H3	0.7215	0.1945	0.6336	0.118*	
C4	0.5062 (4)	0.2564 (4)	0.58242 (17)	0.0920 (9)	
H4	0.4537	0.2952	0.6298	0.110*	
C5	0.4199 (4)	0.2573 (4)	0.51124 (19)	0.0991 (10)	
H5	0.3093	0.2923	0.5115	0.119*	
C6	0.4985 (3)	0.2060 (3)	0.43989 (15)	0.0781 (7)	
H6	0.4420	0.2053	0.3916	0.094*	
C7	0.6425 (2)	0.3393 (2)	0.23941 (10)	0.0408 (4)	
C8	0.6569 (2)	0.4934 (2)	0.22907 (10)	0.0424 (4)	
C9	0.7938 (2)	0.5131 (2)	0.27424 (11)	0.0439 (4)	
C10	0.8617 (3)	0.6446 (2)	0.28430 (14)	0.0584 (5)	
H10	0.8179	0.7435	0.2589	0.070*	
C11	0.9950 (3)	0.6244 (3)	0.33266 (18)	0.0738 (7)	
H11	1.0405	0.7113	0.3412	0.089*	
C12	1.0626 (3)	0.4773 (3)	0.36883 (18)	0.0771 (7)	
H12	1.1536	0.4675	0.4008	0.093*	
C13	0.9999 (3)	0.3449 (3)	0.35916 (15)	0.0625 (5)	
H13	1.0466	0.2461	0.3836	0.075*	
C14	0.8635 (2)	0.3653 (2)	0.31128 (11)	0.0440 (4)	
C15	0.5596 (2)	0.6261 (2)	0.17657 (12)	0.0516 (5)	
C16	0.5615 (3)	0.6198 (3)	0.08490 (12)	0.0593 (5)	
H16A	0.5066	0.5359	0.0729	0.071*	
H16B	0.6722	0.5927	0.0657	0.071*	
C17	0.4848 (4)	0.7702 (3)	0.03593 (16)	0.0880 (8)	
H17A	0.3710	0.7884	0.0469	0.132*	
H17B	0.5053	0.7613	−0.0226	0.132*	
H17C	0.5291	0.8566	0.0523	0.132*	
C18	0.5229 (2)	0.2611 (2)	0.20724 (10)	0.0442 (4)	
H18	0.5563	0.1621	0.1884	0.053*	
C19	0.3680 (2)	0.3249 (2)	0.20359 (11)	0.0467 (4)	
H19	0.3352	0.4250	0.2212	0.056*	
C20	0.2462 (2)	0.2449 (2)	0.17303 (11)	0.0453 (4)	
C21	0.2582 (3)	0.0828 (2)	0.19095 (14)	0.0566 (5)	
H21	0.3466	0.0271	0.2201	0.068*	
C22	0.1449 (3)	0.0018 (2)	0.16730 (14)	0.0589 (5)	
H22	0.1580	−0.1070	0.1796	0.071*	
C23	0.0107 (2)	0.0832 (2)	0.12499 (12)	0.0518 (4)	
C24	−0.0071 (2)	0.2427 (2)	0.10536 (12)	0.0487 (4)	
H23	−0.0963	0.2976	0.0764	0.058*	
C25	0.1097 (2)	0.3207 (2)	0.12932 (11)	0.0452 (4)	
N1	0.77198 (17)	0.25460 (17)	0.28902 (9)	0.0427 (3)	
N3	0.0859 (2)	0.4903 (2)	0.10290 (11)	0.0554 (4)	
O1	0.6646 (2)	0.00599 (16)	0.31000 (9)	0.0651 (4)	
O2	0.92318 (19)	0.02047 (18)	0.37066 (10)	0.0682 (4)	
O3	0.4897 (3)	0.7364 (2)	0.20882 (12)	0.1283 (11)	
O5	−0.04694 (18)	0.55877 (18)	0.08461 (11)	0.0692 (4)	
O6	0.2012 (2)	0.5539 (2)	0.09605 (17)	0.1115 (9)	
S1	0.76175 (6)	0.08992 (5)	0.35118 (3)	0.04869 (14)	
Cl1	−0.13374 (7)	−0.01698 (7)	0.09611 (4)	0.07319 (19)	

### Atomic displacement parameters (Å^2^)

**Table:**

	*U*^11^	*U*^22^	*U*^33^	*U*^12^	*U*^13^	*U*^23^
C1	0.0570 (11)	0.0502 (10)	0.0409 (9)	−0.0105 (8)	−0.0042 (8)	0.0040 (7)
C2	0.0641 (14)	0.121 (2)	0.0511 (12)	−0.0321 (14)	−0.0066 (10)	−0.0076 (13)
C3	0.098 (2)	0.169 (3)	0.0489 (13)	−0.071 (2)	0.0058 (13)	−0.0239 (17)
C4	0.114 (3)	0.112 (2)	0.0570 (15)	−0.0362 (19)	0.0260 (16)	−0.0210 (15)
C5	0.0807 (18)	0.127 (3)	0.0700 (17)	0.0174 (18)	0.0207 (14)	0.0128 (17)
C6	0.0669 (14)	0.106 (2)	0.0486 (12)	0.0085 (14)	−0.0037 (10)	0.0089 (12)
C7	0.0405 (8)	0.0465 (9)	0.0314 (7)	0.0034 (7)	−0.0039 (6)	−0.0038 (6)
C8	0.0444 (9)	0.0458 (9)	0.0330 (8)	0.0039 (7)	−0.0042 (7)	−0.0044 (7)
C9	0.0421 (9)	0.0493 (9)	0.0373 (8)	−0.0004 (7)	−0.0008 (7)	−0.0048 (7)
C10	0.0591 (12)	0.0499 (11)	0.0654 (13)	−0.0074 (9)	−0.0074 (10)	−0.0043 (9)
C11	0.0639 (14)	0.0660 (14)	0.0959 (18)	−0.0182 (11)	−0.0190 (13)	−0.0110 (13)
C12	0.0568 (13)	0.0819 (17)	0.0943 (19)	−0.0116 (12)	−0.0320 (13)	−0.0059 (14)
C13	0.0503 (11)	0.0655 (13)	0.0677 (13)	0.0003 (9)	−0.0196 (10)	0.0001 (10)
C14	0.0398 (8)	0.0495 (9)	0.0400 (9)	−0.0013 (7)	−0.0029 (7)	−0.0035 (7)
C15	0.0597 (11)	0.0460 (10)	0.0443 (10)	0.0046 (8)	−0.0140 (8)	−0.0024 (8)
C16	0.0538 (11)	0.0768 (14)	0.0393 (9)	0.0031 (10)	−0.0010 (8)	0.0057 (9)
C17	0.117 (2)	0.0850 (18)	0.0532 (13)	−0.0059 (16)	−0.0168 (14)	0.0213 (12)
C18	0.0476 (9)	0.0459 (9)	0.0369 (8)	−0.0009 (7)	−0.0060 (7)	−0.0045 (7)
C19	0.0471 (9)	0.0481 (10)	0.0424 (9)	−0.0022 (8)	−0.0022 (7)	−0.0042 (7)
C20	0.0412 (9)	0.0489 (10)	0.0430 (9)	−0.0024 (7)	0.0006 (7)	−0.0014 (7)
C21	0.0533 (11)	0.0505 (11)	0.0612 (12)	−0.0003 (9)	−0.0098 (9)	0.0045 (9)
C22	0.0625 (12)	0.0478 (10)	0.0644 (13)	−0.0080 (9)	−0.0033 (10)	0.0017 (9)
C23	0.0503 (10)	0.0574 (11)	0.0486 (10)	−0.0131 (9)	0.0045 (8)	−0.0052 (8)
C24	0.0388 (9)	0.0563 (11)	0.0482 (10)	−0.0044 (8)	0.0006 (7)	−0.0001 (8)
C25	0.0395 (9)	0.0479 (9)	0.0447 (9)	−0.0028 (7)	0.0035 (7)	0.0015 (7)
N1	0.0420 (7)	0.0436 (8)	0.0389 (7)	0.0004 (6)	−0.0064 (6)	0.0001 (6)
N3	0.0441 (8)	0.0534 (9)	0.0655 (10)	−0.0064 (7)	−0.0053 (7)	0.0068 (8)
O1	0.0916 (11)	0.0455 (7)	0.0589 (9)	−0.0115 (7)	−0.0143 (8)	−0.0052 (6)
O2	0.0624 (9)	0.0575 (8)	0.0722 (10)	0.0174 (7)	−0.0096 (7)	0.0063 (7)
O3	0.205 (3)	0.0840 (13)	0.0636 (11)	0.0794 (16)	−0.0502 (14)	−0.0264 (10)
O5	0.0509 (8)	0.0593 (9)	0.0903 (12)	0.0050 (7)	−0.0109 (8)	0.0038 (8)
O6	0.0654 (11)	0.0756 (12)	0.189 (2)	−0.0286 (9)	−0.0420 (13)	0.0514 (14)
S1	0.0554 (3)	0.0407 (2)	0.0451 (2)	0.00326 (19)	−0.00822 (19)	0.00039 (18)
Cl1	0.0676 (4)	0.0741 (4)	0.0840 (4)	−0.0276 (3)	−0.0047 (3)	−0.0088 (3)

### Geometric parameters (Å, º)

**Table:**

C1—C6	1.374 (3)	C15—O3	1.185 (2)
C1—C2	1.380 (3)	C15—C16	1.483 (3)
C1—S1	1.747 (2)	C16—C17	1.506 (3)
C2—C3	1.373 (4)	C16—H16A	0.9700
C2—H2	0.9300	C16—H16B	0.9700
C3—C4	1.355 (5)	C17—H17A	0.9600
C3—H3	0.9300	C17—H17B	0.9600
C4—C5	1.383 (5)	C17—H17C	0.9600
C4—H4	0.9300	C18—C19	1.329 (3)
C5—C6	1.378 (4)	C18—H18	0.9300
C5—H5	0.9300	C19—C20	1.468 (3)
C6—H6	0.9300	C19—H19	0.9300
C7—C8	1.355 (3)	C20—C21	1.391 (3)
C7—N1	1.427 (2)	C20—C25	1.399 (2)
C7—C18	1.454 (2)	C21—C22	1.373 (3)
C8—C9	1.437 (2)	C21—H21	0.9300
C8—C15	1.493 (2)	C22—C23	1.387 (3)
C9—C10	1.393 (3)	C22—H22	0.9300
C9—C14	1.394 (2)	C23—C24	1.369 (3)
C10—C11	1.371 (3)	C23—Cl1	1.725 (2)
C10—H10	0.9300	C24—C25	1.382 (3)
C11—C12	1.379 (4)	C24—H23	0.9300
C11—H11	0.9300	C25—N3	1.468 (2)
C12—C13	1.373 (3)	N1—S1	1.6720 (15)
C12—H12	0.9300	N3—O6	1.202 (2)
C13—C14	1.388 (3)	N3—O5	1.205 (2)
C13—H13	0.9300	O1—S1	1.4153 (16)
C14—N1	1.418 (2)	O2—S1	1.4230 (15)
			
C6—C1—C2	121.4 (2)	C17—C16—H16A	108.5
C6—C1—S1	118.79 (16)	C15—C16—H16B	108.5
C2—C1—S1	119.75 (18)	C17—C16—H16B	108.5
C3—C2—C1	119.1 (3)	H16A—C16—H16B	107.5
C3—C2—H2	120.5	C16—C17—H17A	109.5
C1—C2—H2	120.5	C16—C17—H17B	109.5
C4—C3—C2	120.0 (3)	H17A—C17—H17B	109.5
C4—C3—H3	120.0	C16—C17—H17C	109.5
C2—C3—H3	120.0	H17A—C17—H17C	109.5
C3—C4—C5	121.1 (3)	H17B—C17—H17C	109.5
C3—C4—H4	119.5	C19—C18—C7	122.80 (17)
C5—C4—H4	119.5	C19—C18—H18	118.6
C6—C5—C4	119.6 (3)	C7—C18—H18	118.6
C6—C5—H5	120.2	C18—C19—C20	123.04 (17)
C4—C5—H5	120.2	C18—C19—H19	118.5
C1—C6—C5	118.7 (2)	C20—C19—H19	118.5
C1—C6—H6	120.6	C21—C20—C25	115.37 (17)
C5—C6—H6	120.6	C21—C20—C19	119.73 (16)
C8—C7—N1	108.46 (15)	C25—C20—C19	124.82 (16)
C8—C7—C18	129.63 (15)	C22—C21—C20	122.68 (18)
N1—C7—C18	121.90 (15)	C22—C21—H21	118.7
C7—C8—C9	108.69 (15)	C20—C21—H21	118.7
C7—C8—C15	128.84 (16)	C21—C22—C23	119.55 (19)
C9—C8—C15	122.37 (17)	C21—C22—H22	120.2
C10—C9—C14	119.98 (17)	C23—C22—H22	120.2
C10—C9—C8	132.32 (17)	C24—C23—C22	120.32 (19)
C14—C9—C8	107.70 (16)	C24—C23—Cl1	119.70 (16)
C11—C10—C9	118.2 (2)	C22—C23—Cl1	119.98 (16)
C11—C10—H10	120.9	C23—C24—C25	118.76 (17)
C9—C10—H10	120.9	C23—C24—H23	120.6
C10—C11—C12	121.1 (2)	C25—C24—H23	120.6
C10—C11—H11	119.5	C24—C25—C20	123.31 (17)
C12—C11—H11	119.5	C24—C25—N3	115.63 (15)
C13—C12—C11	122.2 (2)	C20—C25—N3	121.01 (16)
C13—C12—H12	118.9	C14—N1—C7	107.57 (13)
C11—C12—H12	118.9	C14—N1—S1	120.91 (11)
C12—C13—C14	116.9 (2)	C7—N1—S1	123.89 (12)
C12—C13—H13	121.5	O6—N3—O5	122.39 (18)
C14—C13—H13	121.5	O6—N3—C25	118.61 (16)
C13—C14—C9	121.65 (19)	O5—N3—C25	118.88 (16)
C13—C14—N1	130.80 (18)	O1—S1—O2	120.22 (10)
C9—C14—N1	107.55 (14)	O1—S1—N1	106.22 (8)
O3—C15—C16	122.04 (18)	O2—S1—N1	106.01 (9)
O3—C15—C8	119.14 (17)	O1—S1—C1	109.63 (10)
C16—C15—C8	118.71 (16)	O2—S1—C1	109.04 (10)
C15—C16—C17	114.9 (2)	N1—S1—C1	104.52 (8)
C15—C16—H16A	108.5		
			
C6—C1—C2—C3	−1.9 (4)	C25—C20—C21—C22	0.3 (3)
S1—C1—C2—C3	−178.8 (2)	C19—C20—C21—C22	177.18 (19)
C1—C2—C3—C4	−1.3 (5)	C20—C21—C22—C23	−1.1 (3)
C2—C3—C4—C5	3.6 (5)	C21—C22—C23—C24	1.4 (3)
C3—C4—C5—C6	−2.7 (5)	C21—C22—C23—Cl1	−179.18 (17)
C2—C1—C6—C5	2.7 (4)	C22—C23—C24—C25	−0.7 (3)
S1—C1—C6—C5	179.7 (2)	Cl1—C23—C24—C25	179.79 (14)
C4—C5—C6—C1	−0.4 (5)	C23—C24—C25—C20	−0.1 (3)
N1—C7—C8—C9	−1.87 (19)	C23—C24—C25—N3	177.42 (17)
C18—C7—C8—C9	179.19 (17)	C21—C20—C25—C24	0.4 (3)
N1—C7—C8—C15	174.39 (17)	C19—C20—C25—C24	−176.37 (17)
C18—C7—C8—C15	−4.6 (3)	C21—C20—C25—N3	−177.05 (18)
C7—C8—C9—C10	179.8 (2)	C19—C20—C25—N3	6.2 (3)
C15—C8—C9—C10	3.2 (3)	C13—C14—N1—C7	179.4 (2)
C7—C8—C9—C14	0.9 (2)	C9—C14—N1—C7	−1.50 (18)
C15—C8—C9—C14	−175.62 (16)	C13—C14—N1—S1	28.8 (3)
C14—C9—C10—C11	−1.5 (3)	C9—C14—N1—S1	−152.11 (13)
C8—C9—C10—C11	179.8 (2)	C8—C7—N1—C14	2.11 (19)
C9—C10—C11—C12	1.6 (4)	C18—C7—N1—C14	−178.85 (15)
C10—C11—C12—C13	−0.7 (4)	C8—C7—N1—S1	151.63 (13)
C11—C12—C13—C14	−0.3 (4)	C18—C7—N1—S1	−29.3 (2)
C12—C13—C14—C9	0.4 (3)	C24—C25—N3—O6	−155.4 (2)
C12—C13—C14—N1	179.3 (2)	C20—C25—N3—O6	22.2 (3)
C10—C9—C14—C13	0.5 (3)	C24—C25—N3—O5	20.7 (3)
C8—C9—C14—C13	179.57 (18)	C20—C25—N3—O5	−161.72 (19)
C10—C9—C14—N1	−178.64 (17)	C14—N1—S1—O1	−178.89 (13)
C8—C9—C14—N1	0.39 (19)	C7—N1—S1—O1	35.43 (16)
C7—C8—C15—O3	121.1 (3)	C14—N1—S1—O2	−49.93 (15)
C9—C8—C15—O3	−63.1 (3)	C7—N1—S1—O2	164.38 (14)
C7—C8—C15—C16	−62.5 (3)	C14—N1—S1—C1	65.22 (15)
C9—C8—C15—C16	113.3 (2)	C7—N1—S1—C1	−80.47 (15)
O3—C15—C16—C17	6.7 (4)	C6—C1—S1—O1	−37.0 (2)
C8—C15—C16—C17	−169.6 (2)	C2—C1—S1—O1	139.98 (19)
C8—C7—C18—C19	−39.8 (3)	C6—C1—S1—O2	−170.50 (19)
N1—C7—C18—C19	141.39 (18)	C2—C1—S1—O2	6.5 (2)
C7—C18—C19—C20	−178.51 (16)	C6—C1—S1—N1	76.5 (2)
C18—C19—C20—C21	37.1 (3)	C2—C1—S1—N1	−106.5 (2)
C18—C19—C20—C25	−146.33 (19)		

### Hydrogen-bond geometry (Å, º)

**Table:**

*D*—H···*A*	*D*—H	H···*A*	*D*···*A*	*D*—H···*A*
C10—H10···O1^i^	0.93	2.60	3.327 (3)	136
C16—H16*B*···O5^ii^	0.97	2.37	3.260 (3)	152

Symmetry codes: (i) *x*, *y*+1, *z*; (ii) *x*+1, *y*, *z*.

## References

[bb1] Bruker (2004). *APEX2* and *SAINT* Bruker AXS Inc., Madison, Wisconsin, USA.

[bb2] Chakkaravarthi, G., Dhayalan, V., Mohanakrishnan, A. K. & Manivannan, V. (2008). *Acta Cryst.* E**64**, o749. 10.1107/S1600536808007678 PMC2960947 21202139

[bb3] Chakkaravarthi, G., Panchatcharam, R., Dhayalan, V., Mohanakrishnan, A. K. & Manivannan, V. (2010). *Acta Cryst.* E**66**, o2957. 10.1107/S160053681004198X PMC3009294 21589125

[bb4] Okabe, N. & Adachi, Y. (1998). *Acta Cryst.* C**54**, 386–387.

[bb5] Sheldrick, G. M. (1996). *SADABS*, University of Göttingen, Germany.

[bb6] Sheldrick, G. M. (2008). *Acta Cryst.* A**64**, 112–122. 10.1107/S0108767307043930 18156677

[bb7] Spek, A. L. (2009). *Acta Cryst.* D**65**, 148–155. 10.1107/S090744490804362X PMC2631630 19171970

[bb8] Srivastava, Anupam & Pandeya, S. N. (2011). *JCPR*, **1**, 1–17.

